# Tenascin-X Deficiency Causing Classical-Like Ehlers-Danlos Syndrome Type 1 in Humans is a Significant Risk Factor of Gastrointestinal and Tracheal Ruptures

**DOI:** 10.14309/ctg.0000000000000821

**Published:** 2025-01-14

**Authors:** Jonneke E. van Gurp, Rosan L. Lechner, Dimitra Micha, Alessandra Maugeri, Eelco Dulfer, Fleur S. van Dijk, Daniel Keszthelyi, Edoardo Malfatti, Akiharu Kubo, Nicol C. Voermans, Serwet Demirdas

**Affiliations:** 1 Department of Clinical Genetics, Erasmus MC, University Medical Center, Rotterdam, the Netherlands;; 2 Department of Human Genetics, Amsterdam Reproduction and Development, Amsterdam Movement Sciences, Amsterdam UMC, Vrije Universiteit Amsterdam, Amsterdam, the Netherlands;; 3 Department of Genetics, University Medical Center Groningen, Groningen, the Netherlands;; 4 National Ehlers Danlos Syndrome Service, London North West University Healthcare NHS Trust, Harrow, London, UK;; 5 Department of Metabolism, Digestion and Reproduction, Section Genomics & Genetics, Imperial, London, UK;; 6 Department of Gastroenterology-Hepatology, Maastricht University Medical Center, Maastricht, the Netherlands;; 7 APHP, Centre de Référence de Pathologie Neuromusculaire Nord-Est-Ile-de-France, Henri Mondor Hospital, University Paris-Est, Créteil, France;; 8 Department of Dermatology, Keio University School of Medicine, Tokyo, Japan;; 9 Department of Neurology, Radboud University Medical Centre, Nijmegen, the Netherlands.

**Keywords:** Ehlers-Danlos syndrome, EDS, classical-like EDS, tenascin-X, TNXB, TNX, gastrointestinal rupture, connective tissue

## Abstract

**INTRODUCTION:**

Classical-like Ehlers-Danlos syndrome type 1 (clEDS1) is a very rare form of Ehlers-Danlos syndrome caused by tenascin-X deficiency, with only 56 individuals reported in medical literature. Tenascin-X is an extracellular matrix protein needed for collagen stability. Previous publications propose that individuals with clEDS1 might be at risk of gastrointestinal (GI) tract perforations and/or tracheal ruptures. The aim of this study was to characterize complications resulting from perforations of the GI tract and/or tracheal rupture in an international case series of individuals with clEDS1 due to disease-related tissue fragility.

**METHODS:**

This case series includes individuals with confirmed clEDS1 and GI perforations and/or tracheal ruptures from participating centers. Researchers who previously reported such individuals were contacted for additional information. A retrospective assessment of clinical features was performed.

**RESULTS:**

Fifteen individuals were included. Ten had spontaneous GI perforations, 7 of whom had multiple GI perforations. Almost all had severe diverticulosis. Three individuals experienced iatrogenic tracheal ruptures.

**DISCUSSION:**

Severe GI complications, such as perforation, and tracheal rupture were observed in a substantial number of individuals with clEDS1. As these features seem significantly more common in clEDS1 than in the average population, we advise vigilance during intubation and GI endoscopic interventions of individuals with clEDS1. Routine referrals to clinical geneticists are recommended for patients with symptoms indicative of clEDS1, especially with unexplained GI perforations and connective tissue symptoms. Our findings offer valuable insights for the clinical management of clEDS1 and underscore the importance of specialized care, providing a foundation for improved clinical guidelines and preventive strategies.

## Graphical abstract

**Figure:**
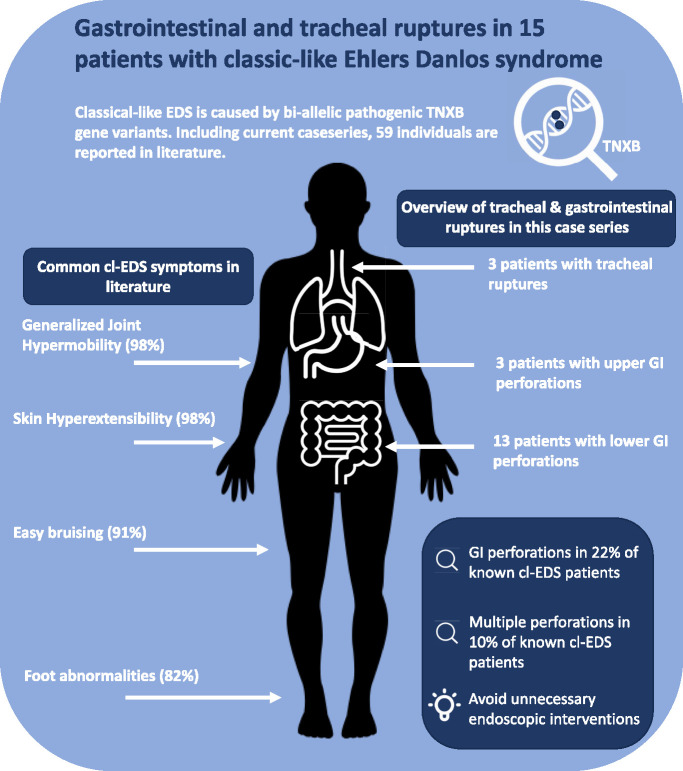

## INTRODUCTION

Classical-like Ehlers-Danlos syndrome type 1 (clEDS1) is one of the 13 types of Ehlers-Danlos syndrome (EDS), a very rare form of this heterogeneous group of connective tissue disorders (1,2). Currently, 56 individuals with clEDS1 have been reported in the literature (3). clEDS1 is caused by tenascin-X (TNX) deficiency because of biallelic pathogenic *TNXB* gene variants (1,4). TNX is a glycoprotein that is thought to mediate interaction between cells and the extracellular matrix by binding directly to fibrillary collagen through decorin and to integrin on cell surfaces. Furthermore, it may play a role in fibrillogenesis through interaction with collagen XII and collagen VI (5). Mao et al (6) showed that deficiency of TNX leads to a reduction in collagen deposition, which may account for the weakening of the extracellular matrix (6). Individuals diagnosed with clEDS1 have been previously reported with gastrointestinal (GI) complications, such as diverticulosis, gastric ulcers, spontaneous perforation of the intestines, and tracheal ruptures (2,7–9). Both spontaneous and iatrogenic perforations have been described. These complications are detrimental to the individual. They can even lead to sepsis and death (2,8). Severe GI symptoms are associated with other types of EDS, but not typically with clEDS1. However, current reports suggest that it may be a much more frequent complication for individuals with clEDS1 than previously considered. Bearing in mind the severe risk associated with GI fragility, timely intervention can prevent life-threatening complications (2,8). Thus, an adequate overview of the nature and severity of GI symptoms in clEDS1 is essential. Knowledge and awareness of GI complications and tracheal ruptures are expected to improve treatment, perioperative management, and follow-up (10). Prevention and early diagnosis can ultimately deter a potentially life-threatening course (11). Some previous studies have briefly described GI complications in patients with clEDS1. To address the critical knowledge gap of these complications, we made a more extensive characterization of GI complications and/or tracheal ruptures in a case series and literature review of individuals with clEDS1. This extensive overview was made to provide adequate risk assessment as a foundation for improving clinical guidelines and follow-up.

## METHODS

### Patient selection

The aim of this case series and literature review was to give a retrospective overview of individuals with both clEDS1 and GI and/or tracheal fragility. A literature search was performed in PubMed in September 2021 and updated in February 2024. The following search term was used: tenascin X deficiency [All Fields] OR (“Ehlers-Danlos syndrome caused by tenascin X deficiency” [MeSH Terms] OR “classical like EDS” [All Fields] OR “TNX deficient ehlers danlos” [All Fields])) OR (“tenascin X” [MeSH Terms] OR (“tenascin X” [All Fields]) OR “clEDS”[All Fields] OR “classical-like ehlers danlos syndrome” [All Fields]. Articles were reviewed and selected if individuals were reported to have GI complications and/or tracheal rupture. The authors of already published individuals with these severe complications were contacted to contribute all available additional clinical information. Furthermore, experts in the field of clEDS1 were contacted. Additional individuals with clEDS1 were identified by their clinicians, and the data were collected through collaborations with the participating centers (Erasmus Medical Centre, Rotterdam; Radboud University Medical Centre, Nijmegen; Maastricht University Medical Centre, Maastricht; University Medical Centre Groningen, Groningen; National EDS service London; Hôpital La Pitié Salpêtrière, Paris; Keio University, Tokyo).

The individuals included were confirmed to have TNX-deficiency either by quantification of the protein levels in blood by ELISA analysis and/or identification of biallelic (likely) pathogenic variants in the *TNXB* gene. The second inclusion criterion was a history of GI rupture or tracheal rupture. Individuals were excluded when medical information about the GI complications and clEDS1 diagnosis was unclear. The study was conducted in compliance with the Declaration of Helsinki and approved by the Institutional Review Board of the Erasmus Medical Center (MEC-2021-0536) and Maastricht University Medical Center (METC 2021-2947).

### Clinical features

To enhance knowledge about the clinical phenotype and natural history of clEDS1 and, in particular, the GI/tracheal complications, clinical characteristics of clEDS1 individuals with GI complications were retrieved from individuals' records. Information about sex, age of diagnosis, symptoms at diagnosis, features compatible with the criteria for clEDS1 (3), family history, genotype, and TNX serum levels were obtained. To assess the GI fragility in these individuals, information was collected about the occurrence of GI perforation, GI intestinal disease, vascular disease, and other risk factors associated with GI perforation, such as herniation, smoking, cytomegalovirus infection, and indomethacin use.

### Statistical analysis

Owing to the limited number of individuals with this rare disease, primarily descriptive analyses were performed to assess the relation between the phenotype, genotype, individual characteristics, and the type of GI complication or rupture of the individuals. Dichotomous data are expressed as a number and percentage. Continuous data are expressed as median and min-max. Distribution of data was visually assessed using histograms and a QQ plot. Owing to small numbers and skewing of data, nonparametric tests were used for statistical analysis. Continuous data were evaluated using the Wilcoxon Mann-Whitney *U* test or the Kruskal-Wallis test. Categorical data were assessed using the χ^2^ test or Fisher exact test. Time to the first perforation was examined using a Kaplan-Meier curve. The Breslow test was used to assess the significance. All tests are 2-sided, and the significance was assumed at *P* value < 0.05. Statistical analysis was performed using SPSS software version 22 (IBM Corp. Released 2013; IBM SPSS Statistics for Windows, Version 22.0. Armonk, NY).

## RESULTS

### Study population and characteristics

In this study, we included 15 individuals, of which 12 have been published previously (2,4,7–9,12–14). The general individual characteristics and genotype of all 15 individuals are presented in Table 1. In our group, 11 individuals were female (73%). Most individuals (53%) developed the first symptom associated with clEDS1 in childhood. The median age at diagnosis was 46 years, varying between 22 and 69 years. The symptoms that led to diagnosis were reported in 5 of our patients. Multiple joint dislocations and increased bleeding tendency were most common. The median age at the moment of inclusion was 57 years.

**Table 1.** General patient characteristics of 15 clEDS patients with gastrointestinal or tracheal perforations

	Patient 1	Patient 2	Patient 3	Patient 4	Patient 5	Patient 6	Patient 7	Patient 8	Patient 9	Patient 10	Patient 11	Patient 12	Patient 13	Patient 14	Patient 15
Sex	Male	Female	Female	Female	Female	Female	Male	Female	Female	Female	Female	Female	Male	Female	Male
Age at inclusion	71 yr	44 yr	57 yr	NA	61 yr	50 yr	77 yr	56 yr	49 yr	52 yr	53 yr	65 yr	53 yr	45 yr	69 yr
Age at first complication	55 yr	40 yr	51 yr	NA	10 mo	48 yr	Childhood	Childhood	Childhood	Early infancy	Childhood	Childhood	34 yr	Early infancy	52 yr
First symptoms or complications	Gastric problems and bloating	Gastric problems: Bloating, nausea, slow gut movement	NA	NA	Unilateral congenital hip dislocation	Spontaneous perforation of jejunum	Hemarthrosis of knee joint	Hematomas and (sub)luxations	Multiple sprains & dislocation 5th finger	Herniated intestine	Multiple sprains	Hematomas and (sub)luxations	Spontaneous perforation of colonic diverticulum	Congenital hipdislocation	Diverticulitis
Symptoms that led to diagnosis	NA	Rheumatologist: Hypermobility, multiple bruises, and very stretchy and doughy skin	NA	NA	NA	NA	Hemarthrosis of his knee joint	NA	NA	Diagnosed by exome sequencing other symptoms were congenital hip dislocation (operated repeatedly during age 2–6), repeated shoulder dislocation, Sigmoid colon perforation (age 40), intestinal ileus and duodenal perforation via ileus tube, and periodontitis	Multiple heavy bleeds; increased bleeding tendency	NA	NA	Multiple joint dislocations	NA
Age at diagnosis	68 yr	40 yr	NA	49 yr	60 yr	49 yr	46 yr	46 yr	45 yr	45 yr	46 yr	22 yr	37 yr	41 yr	69 yr
Family history of clEDS	NA	2 affected siblings, molecularly confirmed	Affected brother, not molecularly confirmed as died	NA		No	4 daughters TNXB haploinsufficiency; sister has 5 children of whom 2 are likely having EDS	NA	Younger brother multiple sprains and hypermobility, not molecularly confirmed	Sister and niece mild hyperelasticity, not molecularly confirmed	NA	Clinically affected siblings, all TNXB deficient	Brother same type of skin and joint hypermobility	Multiple joint dislocations	Unknown
Allelic status/	Homozygous	Homozygous	Compound heterozygous	Homozygous	Homozygous	Homozygous	Homozygous	Compound heterozygous	Compound heterozygous	Compound heterozygous	Compound heterozygous	Homozygous		Homozygous	Homozygous
Genotype															
Variant c.DNA	c.12174C>G	c.4129G>T	c.8488del/c.10459C>T	c.12174C>G	Chr17(GRCh37:g.32012493_32010952)	c.11479C>T	** **c.2116_2117dup	c.11435_11524 + 30del/c.12174C>G *	c.7826–1G>C/c.9998dupA	c.2539C>T/c.3574C>T (NM_019105.6)	c.12553C>T/c.2590C>T	c.3290_3291del		c.5362del	c.107_108delinsA
Amino acid	p.Cys4058Trp	p.Glu1377*	p.Gln2830fs/p.Gln3487*	p.Cys4058Trp	This concerns an in-frame deletion of 5 exons within the fibronectin III domain	p.Arg3827*	** **p.(Glu707*)	p.?/p.Cys4058Trp	p.?/p.(Asn3333Lysfs∗35)	(p.Arg847*)/(p.Gln1192*)	p.(Arg4185*)c/p.(Gln864*)b	p.(Lys1097Argfs*48)		p.(Thr1788Profs*100)	p.(Ala36Aspfs*68)
Nomenclature in MANE select (NM_001365276.2)	c.12180C>G, p.(Cys4060Trp)	c.4129G>T, p.(Glu1377*)	c.8494del, p.(Gln2832Lysfs*19)/c.10459C>T, p.(Gln3487*)	c.12180C>G, p.(Cys4060Trp)		c.11485C>T, p.(Arg3829*)	c.2116_2117dup, p.(Glu707*)	Unknown	c.7826-1G>C, p.?/c.10004dup, p.(Asn3335Lysfs*35)	c.2539C>T, p.(Arg847*)/c.3574C>T,p.(Gln1192*)	c.12559C>T,p.(Arg4187*)/c.2590C>T, p.(Gln864*)	c.3290_3291del, p.(Lys1097Argfs*48)		c.5362del,p.(Thr1788Profs*100)	c.107_108delinsA, p.(Ala36Aspfs*68)
Exon	40	11	resp. exon 25/31	40	33–37	35	Exon 3		Intron 22/exon 29	Exon 6/Exon 9	Exon 43/Exon 6	Exon 8		Exon 15	Exon 2
Other genetic findings	NA	NA	NA	NA	NA	None	NA	Carrier of adrenogenital syndrome	Other findings: 700Kb (47,392,150_48,100,155, hg19) in the 21q22.3 cytoband	NA	NA	NA	NA	NA	NA

clEDS1, classical-like Ehlers-Danlos syndrome type 1; NA, not available; TNXB, tenascin-X coding gene.

Most individuals (85%, 11/13) had at least one (suspected) truncating variant or null variant. The Cys4060Trp variant was reported 5 times in 3 individuals from different countries. There is no known familial connection between these individuals.

The general clEDS1-related symptoms that were identified in the individuals are provided in Supplemental Table 1 (http://links.lww.com/CTG/B267), which also contains information of previous reports of these individuals. Skin hyperextensibility was seen in 13 of 15 individuals (87%). The Beighton score was reported in 13 individuals. The median Beighton score was 4 (out of 9), ranging from 1 to 9. Either general joint hypermobility or a Beighton score ≥5 was reported in 8 of our individuals (53%). Fragility of the skin was reported in 8 individuals, and in 6 individuals (40%), delayed wound healing was observed.

**Figure 1.**
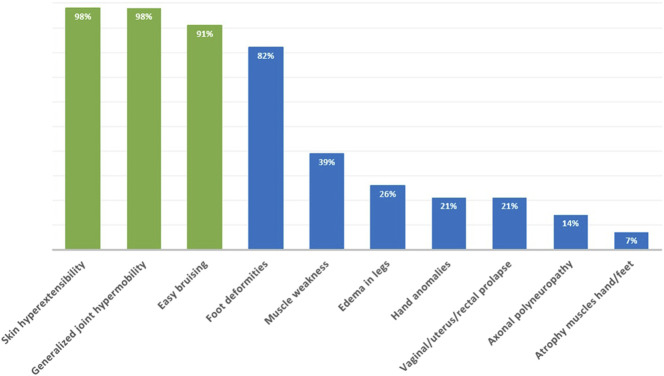
Clinical manifestations of clEDS type 1 in 59 clEDS individuals. Green represents major criteria of clEDS, blue represents minor criteria. Cohort exists of 56 previously reported individuals and 3 newly reported individuals. (3). clEDS1, classical-like Ehlers-Danlos syndrome type 1.

A summary of the clinical manifestations in all reported patients with clEDS type 1 is given in Figure 1.

### Clinical characteristics

**Figure 2.**
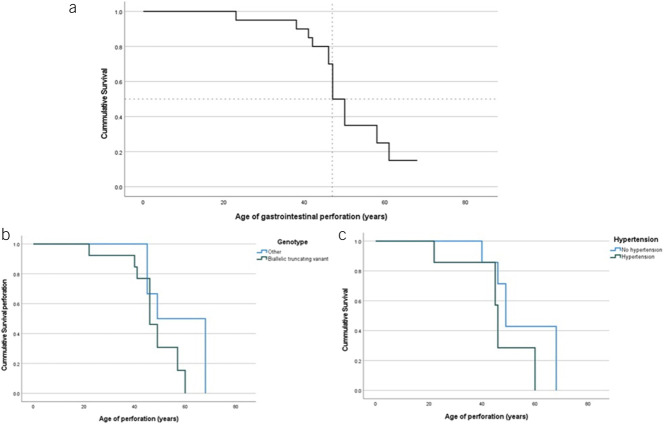
Survival in years to moment of perforation for 20 perforations in patients with 15 classical-like Ehlers-Danlos syndrome.

We present an overview of the GI complications and tracheal ruptures in 15 individuals with clEDS1 in Table 2. Various GI complications were reported, including (spontaneous) perforations. The perforations occurred throughout the entire GI tract. All GI perforations occurred after the age of 20 years, and half of perforations occurred before the age of 40 years (Figure 2a). In 7 of 15 (46%), the cl-EDS diagnosis was known before the first perforation. No significant difference could be seen regarding the age of diagnosis (median age of 47 vs 57 years, *P* = 0.170) and the median age of perforation (49 years vs 41 years, *P* = 0.094).

**Table 2.** Clinical characteristics of 15 clEDS patients with gastrointestinal or tracheal perforations

	Patient 1	Patient 2	Patient 3	Patient 4	Patient 5	Patient 6	Patient 7	Patient 8	Patient 9	Patient 10	Patient 11	Patient 12	Patient 13	Patient 14	Patient 15
Gastrointestinal rupture or perforation	Esophageal rupture during gastroscopy intervention, spontaneous small bowel rupture, small bowel perforation after nasojejunal barium study	Jejunal perforation, possibly due to mural inflammation	Spontaneous transverse colon perforation, small bowel rupture	−	2x Stomach perforation (after taking NSAID and after an injection with NSAID) Spontaneous duodenum perforation	Spontaneous jejunum perforation, jejunum resection; Bowel obstruction due to adhesions and secondary bowel perforation, several resection	−	Spontaneous small bowel perforation	Colonic perforation during colonoscopy	Spontaneous diverticular perforation of the sigmoid colon, followed by incarcerated hernia at the drain tube insertion site and ileus after stoma closure; spontaneous ileus, followed by duodenal perforation (likely due to ileus tube insertion)	Sepsis after duodenal perforation due to nasogastric tube	Spontaneous jejunal perforation possibly due to diverticula	Spontaneous perforation of a colonic diverticulum; multiple intra-abdominal abscesses; second perforation (small bowel) after partial colectomy	Spontaneous perforation of small bowel	1. Perforation of the esophagus due to gastric tube, 2. small bowel perforation due to food impaction, 3. spontaneous small bowel perforation (duodenum), 4. Secondary jejunum perforation due to jejunostomy tube
Age at perforation	55 yr (esophageal) 56 yr (small bowel) 59 yr (second small bowel)	40 yr	51 yr (colon) 3 d later small bowel		55 yr (duodenum and trachea)	47 yr (jejunum) 48 yr (sec. Bowel perforation)	57 yr (tracheal rupture)	62 yr	38 yr	40 yr 45 yr (second perforation)	NA	64 yr	36 yr	42 yr	54 yr (1&2) 65 yr (3) 69 yr (4)
Diverticulitis	−	NA	NA	−	+	+	NA	+	−	+	+	NA	+	NA	+
Diverticulosis	Pan-colonic diverticulosis	−	−	+	+	+	NA	+	+	+	+	+, Jejunum, sigmoid	+, Duodenum, sigmoid	+	+, Duodenum
Obstipation	+	+	NA	NA	NA	+	NA	NA	+	+	+	NA	NA	NA	NA
Other GI disease	−	Slow gut motility; significant bloating, nausea, vomiting	−	GI bleeding	IBS	Stenotic anastomosis with skin fistulizing to skin. Bowel stenosis obstruction permanent gastrostomy	Gastric hemorrhage due to ulcers	Peritonitis caused by small bowel perforation; small bowel ileus	−	Both spontaneous and secondary gastrointestinal ileus during her 40s	Gastric ulcer, GI bleed; hemorrhoids	−	−	Recurrent aphthous stomatitis, ileitis	Ileus at 58 yr
Unexplained abdominal pain	+	+	NA	NA	+	−	NA	NA	+	+	NA	NA	NA	NA	+
Other patients in family with GI complication	−	−	Brother: Jejunal perforation, followed by sigmoid colon perforation; rectal prolapse	−	NA	−	NA	NA	−	NA	−	NA	−	−	NA
Tracheal rupture	−	−	NA	NA	With intubation, significant bleeding occurred	−	Sinus piriformis perforation by transesophageal ultrasound probe	Iatrogenic pharynx-mediastinum perforation caused by a fausse route of nasogastric tube	−	−	−	−	−	−	NA

clEDS1, classical-like Ehlers-Danlos syndrome type 1; GI, gastrointestinal; IBS, irritable bowel syndrome; NA, not available; NSAID, nonsteroidal anti-inflammatory drug.

In individuals where the clEDS was diagnosed first, perforation occurred 11 years (1–42 years) after diagnosis. In 2 patients with a known cl-EDS diagnosis, the perforation occurred as a complication to an intervention (gastroscopy and colonoscopy).

Ten of the 15 individuals (67%) had spontaneous perforations, median age 51 years, ranging from 36 to 65 years. Perforations occurred in the small bowel (n = 5), duodenum (n = 1), jejunum (n = 2), and colon (n = 3). Three individuals had multiple spontaneous perforations (patient 3, 6, and 10).

In the case of a nonspontaneous perforation, the perforation was secondary to either an intervention (n = 7) or another predisposing condition (n = 6). Nonspontaneous perforations due to an intervention were seen in 5 individuals. Individual 1 had an esophageal perforation caused by a gastroscopic intervention (age 55 years), and a small bowel perforation occurred after a nasojejunal barium study (age 59 years). In individual 9, a perforation of the colon was caused by a colonoscopy (age 38 years). Individual 10 had a duodenal perforation due to a spontaneous ileus, likely due to ileus tube insertion (45 years old). Individual 11 had a duodenal perforation causing sepsis due to a nasogastric tube (age unknown). Individual 15 had an esophageal perforation due to a gastric tube at the age of 54 years and a jejunum perforation due to a jejunostomy tube at the age of 69 years. Nonspontaneous perforations due to other predisposing factors than solely connective tissue fragility were observed in 5 individuals. Individual 2 had a jejunal perforation, possibly due to mural inflammation, which caused a mesenteric abscess (age 40 years). Individual 5 had 2 stomach tears, both after using a nonsteroidal anti-inflammatory drug (before the age of 55 years). In individual 6, a bowel obstruction caused a secondary bowel perforation with several resections (48 years). Individual 13 first had a spontaneous perforation of a colonic diverticulum at the age of 36 years. A partial colectomy was performed, and 2 weeks later, he had a small bowel perforation. Individual 15 had a small bowel perforation due to food impaction (54 years).

Biallelic variants were identified in 14 of 15 of the investigated individuals; in patient 13, no molecular data were available. Six individuals (46%) had multiple GI perforations (individuals 1, 3, 5, 6, 10, 13, and 15). Individuals 5 and 8 both had 1 or more GI perforations and a tracheal rupture. There was no clear link between the presence of multiple complications between individuals with biallelic truncating variants compared with the individuals who also carried missense variants (56% vs 67%, *P* = 0.88). Figure 2b shows that perforation seemed to occur slightly earlier in patients with biallelic truncating variants, but this was not significantly different (46 vs 49 years of age, respectively, *P* = 0.617).

### Risk factors

Information about the risk factors of GI disease and perforations is presented in Table 3. Six individuals had hypertension, and 2 had an ischemic stroke. Figure 2c shows median perforation was at the age of 49 years at the moment of perforation in individuals without hypertension compared with 46 years in individuals with hypertension (*P* = 0.286). Only 2 individuals had 2 or more risk factors. In 4 patients, incisional hernias occurred. None of the reported perforations occurred as a result of iatrogenic traumatism during hernia repair surgery.

**Table 3.** Risk factors gastrointestinal disease and perforations in of 15 clEDS patients with gastrointestinal or tracheal perforations

	Patient 1	Patient 2	Patient 3	Patient 4	Patient 5	Patient 6	Patient 7	Patient 8	Patient 9	Patient 10	Patient 11	Patient 12	Patient 13	Patient 14	Patient 15
Smoking	—	—	NA	NA	NA	—	NA	NA	—	—	+	NA	—	NA	NA
CVA/TIA	—	—	NA	NA	NA	—	Minor ischemic stroke, left (with speech disorder, paresis of leg and arm)		—	—	Multiple small cortical infarcts frontal lobe right, parietal lobe left	—	—	NA	—
Peripheral vascular disease	NA	—	NA	NA	NA	—	NA	NA	—	—	—	—	NA	NA	—
Hypercholesterolemia	—	—	NA	NA	NA	—	NA	NA	—	—	—	NA	NA	NA	—
Hypertension	—	—	NA	NA	+	—	NA	—	+	+	+	+	NA	NA	+
Hernia	Incisional hernia	Incisional hernia	NA	NA	—	Incisional hernia	NA	NA	—	Incisional hernia	—	—	—	+	—
Coeliac disease	—	—	—	NA	—	—	NA	NA	—	—	—	—	—	—	—
Indomethacin use	—	—	—	NA	NA	—	NA	NA	—	—	—	—	NA	NA	—
Cancer and use of chemotherapy	—	—	—	NA	—	—	NA	Melanoma right leg (2003)	—	—	—	—	NA	NA	—
CMV infection	—	—	—	NA	—	—	NA	NA	—	—	—	NA	NA	NA	—
Inflammatory bowel disease	—	—	—	NA	—	—	NA	NA	—	—	—	—	—	—	—

clEDS1, classical-like Ehlers-Danlos syndrome type 1; CMV, cytomegalovirus; CVA, cerebrovascular accident; NA, not available; TIA, transient ischemic attack.

#### Other GI complications.

Almost all individuals (12 of our 15 individuals) had diverticulosis, of which 4 (patients 1, 12, 13, and 15) had diverticulosis outside of the left-sided colon. In individual 1, a pan-colonic diverticulosis was seen. Individual 12 had multiple jejunum diverticula. In individual 13, duodenal diverticula were observed. Individual 15 had duodenal diverticulosis. Obstipation was common; 6 of the 15 individuals were known to have obstipation. Ten of the individuals also had other GI diseases, such as irritable bowel syndrome, ileus, ileitis, and GI bleeding. In 3 individuals (individuals 4, 7, and 11), GI bleeding was observed (Table 2). Unexplained abdominal pain was reported in 6 individuals. Only individual 3 was known to have a family member with GI complications. Her brother died due to a perforation of his jejunum, followed by a perforation of the sigmoid colon. He also had a rectal prolapse. clEDS1 was plausible in this individual but not confirmed (9).

#### Tracheal rupture.

In 3 of our individuals, tracheal rupture was seen. All individuals had GI complications. Only individual 7 did not have a GI perforation. This individual did have gastric hemorrhage due to ulcers. The tracheal rupture was caused by an intervention in all 3 individuals. In individual 5, acute surgery was needed because of her duodenal perforation. During intubation, significant bleeding occurred. In individual 7, a sinus piriformis perforation was caused by a transesophageal ultrasound probe. In individual 8, a pharynx-mediastinum perforation occurred, caused by a false route of the nasogastric tube.

#### Vascular anomalies.

We also collected data about vascular abnormalities in our individuals. No conspicuous vascular abnormalities were reported. Six individuals had cardiac disease such as mitral valve or aortic valve regurgitation. Individual 1 had a dilated cardiomyopathy at the age of 62 years. No risk factors for this condition were present.

## DISCUSSION

In this overview, we present 15 individuals with clEDS1 and severe GI complications and/or tracheal rupture. 56 individuals with clEDS1 are known in current literature. In this case series, we report 3 new individuals. In our group, 10 individuals had a spontaneous perforation. Combined with the clEDS1 individuals known in the literature, in total, 16.9% (10/59) had a spontaneous perforation. A nonspontaneous perforation was seen in 9 of our individuals (15.3%, 9/59). In total, 22.0% (13/59) had a GI perforation, and 6 individuals had multiple perforations (10.1%, 6/59). Trachea rupture was seen in 5.1% (3/59). In the general population, spontaneous perforation of the bowel is a very rare condition (15); nonspontaneous perforations, for example, due to colonoscopy or gastroscopy are rare, accounting for 0.2% and 0.04%, respectively (16). To our knowledge, numbers about the incidence of spontaneous perforation are not available. In our study, we only used data from individuals with clEDS1 that were already known in the literature and the data of 3 new individuals with severe GI complications or tracheal rupture (patients 5, 6, and 15 table 1–3). Although this study was not designed to assess an incidence of severe GI complications and tracheal ruptures in clEDS1, the incidences of spontaneous and nonspontaneous perforations seem to be significantly higher in individuals with clEDS1 than in the average population. This is likely due to weakness of GI tissue caused by TNX deficiency. Tracheal perforation, a very uncommon and severe complication, was also observed in 3 of our individuals. In all 3 cases, the perforation was caused by an intervention. Individuals with clEDS1 are therefore at significant risk of spontaneous and iatrogenic GI perforations and iatrogenic tracheal rupture.

We collected information about risk factors of perforation or GI complications such as smoking, coeliac disease, and inflammatory bowel disease. Most of our individuals only had 2 or less risk factors. We did not find any remarkable risk factors in our individuals that could have caused the perforations other than the TNX deficiency. We also did not find a connection between the severity of clEDS1 or other GI complications. No association was found between the Beighton score and the occurrence of GI complications based on our descriptive analysis, when compared with other known individuals.

van Dijk et al (3) reported joint hypermobility and skin hyperextensibility in 100% of reported individuals with clEDS1. Skin hyperextensibility was seen in most of our individuals, but only 47% of individuals reported generalized joint hypermobility. Although no direct comparison was made with individuals without GI perforation, this suggests that severity of other clEDS1 symptoms is not predictive of GI fragility. Comparing the type of genetic variants in our cohort with pathogenic and likely pathogenic variants reported in ClinVar showed that the number of truncating variants had a similar distribution (79% vs 85%) (17). It is important to emphasize that TNX deficiency in affected individuals results in the complete absence of the protein. This deficiency is directly linked to the clinical manifestations of clEDS1. While specific genetic variants may provide further insights into clinical severity, the complete absence of TNX remains the most relevant factor influencing the condition.

In this case series, we report 12 individuals with diverticulosis. An additional nonpublished individual with clEDS1 also presented extensive diverticulosis and a posterior rectocele. The variant of this individual was c.5111_5112del/c.11435_11524+30del, p.Phe1704Cysfs*19/p.? located in exon 14/35. This means that in the clEDS1 population, at least 13 individuals are diagnosed with (severe) diverticulosis (22.0%). Possibly, more individuals with clEDS1 may have undiagnosed diverticulosis, which has not (yet) led to perforation. It is compelling to suggest that the prevalence of colonic, particularly small bowel diverticula, is significantly higher in patients with clEDS1 than in the general population. This indicates not only a greater number of diverticula but also an increased rate of complications, likely linked to the connective tissue abnormalities characteristic of this condition. Determining whether diverticula appear at a younger age is challenging because diverticulosis can remain asymptomatic for an extended period, even if present in younger individuals. Although the incidence of diverticulosis is known to be high in the average population (50% of people older than 60 years have colonic diverticula), perforations are most commonly caused by severe inflammation due to diverticulitis (18,19). In our cohort, some of the individuals (n = 3) had spontaneous perforations out of these diverticula, which warrants clinical awareness. Therefore, patients with noticeable diverticulitis or unexplained perforation AND skin hyperextensibility AND hypermobility AND indications for an autosomal recessive disease need to be referred to a clinical geneticist.

### Limitations

Owing to the retrospective nature of our study, not all preferred clinical information was available, such as more extensive information regarding disease management. In addition, limited data were available for some individuals. Therefore, not all risk factors and symptoms of the disease could be analyzed. Furthermore, we used reported individuals and only approached a select group of physicians treating individuals with clEDS1. Owing to this approach, this case series gives an incomplete overview of the whole clEDS1 population. The limited number of individuals mainly enabled descriptive analyses to assess the relation between the phenotype, individual characteristics, and the type of GI complication in these individuals. Statistical analysis were likely underpowered to determine existing differences. No firm conclusions can therefore be drawn. We recommend to include all known individuals with clEDS1 in specialized centers within Europe in a prospective cohort to better review the risk and management of GI complications in cl-EDS. This will lead to a less-biased overview and a better comparison of symptoms and predictors for GI fragility.

In this study, we have reported 3 new cases and reviewed 12 literature cases, highlighting the GI and tracheal fragility in this rare EDS type. Both spontaneous and nonspontaneous perforations can occur in these individuals. All individuals with clEDS1 should therefore be treated with greater caution especially during interventions within the GI system and intubation. In line with the 2022 GeneReviews (3), we recommend avoidance of invasive procedures unless necessary. Clinicians should be particularly judicious in determining the need for endoscopic examination, opting for it only when no other diagnostic or therapeutic options are available. If an endoscopy is deemed necessary, it is essential to minimize tension on the gut wall by avoiding looping of the endoscope, for example, and to have a surgical team on standby to address any potential complications. When intubating, a flexible scope is preferred to reduce the risk of iatrogenic damage to the trachea (20).However, more research is needed into clinical management of perforations in clEDS1.

We also recommend a low threshold for referral to a gastroenterologist in case of abdominal pain and special attention during general anesthesia. In addition, patients with an extensive diverticulitis or an unexplained perforation who also have skin hyperextensibility or hypermobility need to be referred to a clinical geneticist for further evaluation.

We believe our findings offer valuable insights into the clinical management of clEDS1 for the gastroenterologist and underscore the importance of specialized care for these individuals. The detailed characterization of GI and tracheal complications in our study provides a foundation for developing improved clinical guidelines and preventive strategies and further studies concerning this topic.

## CONFLICTS OF INTEREST

**Guarantor of the article:** Serwet Demirdas, PhD, MD.

**Specific author contributions:** Planning and/or conducting the study by S.D./F.S.D.; collecting and/or interpreting data by all authors; drafting the manuscript all authors. All authors have approved the final draft submitted.

**Financial support:** Open access publication costs for this paper were contributed by the Ehlers Danlos society.

**Potential competing interests:** Dr. S. Demirdas is co-chair of the international working group for the rarer types of EDS, within the EDS society. She has received a honorarium in the past, but not for this research or the research paper.

**IRB approval:** The study was conducted in compliance with the Helsinki declaration and approved by the institutional review board of the Erasmus Medical Centre (MEC-2021-0536) and Maastricht University Medical Centre (METC 2021–2947).

**Study Highlights**

**WHAT IS KNOWN**

✓ Classical-like Ehlers-Danlos syndrome type 1 (clEDS1) is a rare type of Ehlers-Danlos syndrome caused by tenascin-X deficiency due to biallelic pathogenic *TNXB* gene variants.
✓ Gastrointestinal (GI) complications and tracheal ruptures have been reported in clEDS1, though not commonly associated with the condition.
✓ These GI complications can be life-threatening, leading to severe outcomes such as sepsis and death.

**WHAT IS NEW HERE**

✓ We included 15 individuals with clEDS1, highlighting that 67% experienced spontaneous GI perforations and 46% had multiple GI perforations.
✓ Three individuals had tracheal ruptures, all caused by medical interventions.
✓ The incidence of spontaneous and nonspontaneous GI perforations is significantly higher in clEDS1 individuals compared with the general population.
✓ The study emphasizes the need for specialized care and suggests that all individuals with clEDS1 should be managed cautiously during GI interventions and intubation to prevent life-threatening complications.

## Supplementary Material

SUPPLEMENTARY MATERIAL
