# Supplementary material for: Tenascin-X Deficiency Causing Classical-Like Ehlers-Danlos Syndrome Type 1 in Humans is a Significant Risk Factor of Gastrointestinal and Tracheal Ruptures
Source: Clin Transl Gastroenterol. 2025 Jan 14;16(3):e00821. doi: 10.14309/ctg.0000000000000821 (PMC11932583; doi:10.14309/ctg.0000000000000821)
Supplement: SUPPLEMENTARY MATERIAL [file ct9-16-e00821-s001.docx]

**Supplemental Table 1.** Symptoms of clEDS in 15 clEDS patients with perforation of the trachea or the gastrointestinal tract

|  | **Patient 1** | **Patient 2** | **Patient 3** | **Patient 4** | **Patient 5** | **Patient 6** | **Patient 7** | **Patient 8** | **Patient 9** | **Patient 10** | **Patient 11** | **Patient 12** | **Patient 13** | **Patient 14** | **Patient 15** |
| --- | --- | --- | --- | --- | --- | --- | --- | --- | --- | --- | --- | --- | --- | --- | --- |
| **Generalized joint hypermobility** | NA | NA | NA | NA | + | - (only small joints) | - (small joints) | + | - | - | + | + | + | - | NA |
| **Beighton score** | 1/9 | 7/9 | 9/9 | 7/9 | 6/9 | 4/9 | 4/9 | 7/9 | 4/9 | 3/9 | 9/9 | 3/9 | NA | 3/9 | NA |
| **Skin hyperextensibility** | + | + | + | + | + | - | + | + | + | + | + | + | + | + | NA |
| **Tissue fragility** | + | + | NA | NA | + | - | NA | + | + | + | NA | + | - | + | NA |
| **Atrophic scarring** | - | Mild | - | NA | - | - | - | - | - | - | - | - | + | - | NA |
| **Easy bruising** | + | + | + | + | + | + | + | + | + | + | + | + | + | + | + |
| **Complaints delayed healing** | + | + | - | - | + | - | NA | + | + | - | - | - | - | + | NA |
| **Soft, velvety, doughy skin** | - | + | + | + | + | + | + | + | + | + | + | + | + | + | NA |
| **Raynaud disease** | + | - | - | NA | - | - | NA | - | - | - | - | + | NA | NA | NA |
| **Molluscoid pseudotumors** | - | - | NA | NA | + | NA | NA | NA | - | NA | - | NA | - | NA | NA |
| **Subcutaneous spheroids** | - | - | NA | NA | NA | NA | NA | NA | - | NA | - | NA | - | + | NA |
| **Hernia** | Incisional hernia | Incisional hernia | NA | NA | - | Incisional hernia | NA | NA | - | Incisional hernia | - | - | - | +, inguinal, umbilical | NA |
| **Epicanthal folds** | - | - | NA | NA | NA | - | NA | NA | - | NA | - | NA | - | NA | NA |
| **Complications of joint hypermobility (sprains, (sub)luxation, pain, flexible flatfoot)** | Dislocation/subluxation | Dislocation/subluxation | - | Dislocation/subluxation | Dislocation/subluxation | - | Frequent subluxations of shoulders | (Sub)luxation of the shoulder, hip, knee, finger(s), wrist | Multiple sprains, luxation 5th finger | Multiple subluxations hip joints. | (Sub)luxation of the shoulder, ankle, hip, finger(s), toe(s); frequent ankle sprains | + (Sub)luxations/sprains of shoulder, wrist, patella, thumbs, clavicula, hip, knee | - | + dislocations | + Joint pain shoulder |
| **Foot deformities (broad/plump forefoot, brachydactyly with excessive skin, pes planus, hallux valgus, peizogenic papules)** | Brachydactyly, hallux valgus, pes planus, deformed toes | Brachydactyly, pes planus | Brachydactyly | Brachydactyly, pes planus, hallux valgus | Brachydactyly; pes planus | Brachydactyly | NA | Hallux valgus, pes planus | NA | Pes planus, subluxation left 5th toe. | - | +, Brachydactyly, claw toes, pes planus, hallux valgus | - | +, Brachydactyly, broad feet | NA |
| **Oedema in legs (without cardiac failure)** | - | - | - | + | + | - | NA | - | - | - | - | - | NA | + | NA |
| **Mild generalized muscle weakness** | + | + | - | + | + | NA | + | + | + | - | NA | + | NA | + | + |
| **Mild distal muscle weakness** | + | NA | NA | NA | + | - | + | + | + | + | + (feet/ toes) | + | NA | + | NA |
| **Axonal polyneuropahty** | NA | NA | NA | NA | NA | - | NA | - | - | + | + | + | NA | NA | NA |
| **Atrophy of muscles in hands and feet** | NA | NA | NA | NA | + | - | NA | NA | + | - | NA | + | NA | NA | NA |
| **Acrogeric hands, mallet finger(s), clinodactyly, brachydactyly** | - | - | - | - | - | Brachydactyly | NA | - | - | - | Acrogenic hands | +, clinodactyly | - | +, Acrogenia, brachydactyly, broad hands | NA |
| **Vaginal/uterus/rectal prolaps** | - | - | - | - | - | - | NA | Vaginal prolapse | Rectal prolapse | - | Rectal prolapse | Rectal prolapse | - | - | NA |
| **Piezogenic papules feet** | + | + | NA | + | NA | NA | NA | - | + | + | - | - | NA | + | NA |
| **Spontaneous ecchymosis** | - | + | NA | NA | NA | + | NA | + | - | + | + | + | NA | NA | NA |
| **Other findings in PE** | NA | NA | NA | NA | Vaginal rupture | NA | Limited physical fitness, easy fatigability, mild bilateral ptosis | Bursitis, chronic myalgias, chronic arthralgias, painful foot soles, complaints of fatigue, refractive error, frequent subconjunctival haemorrhage | Hyperlaxity of the conjunctiva in both eyes, hyperkeratosis pilaris | Slack-skinned face with prominent eyes and a thin nose, scoliosis | Complaints of fatigue; paraesthesia of the hand and legs/feet; refractive error | Morel-Lavallée lesion | - | - | NA |
| **Vascular abnormalities** | NA | - | NA | Spontaneous left calf hematoma; right arm cephalic vein thrombosis; pulmonary embolism | NA | - | NA | NA | - | - | Varicose veins | - | - | - | Secundary thrombosis basilic vein |
| **Cardiac disease** | Dilated cardiomyopathy at age 62 | - | Mild aortic valve regurgitation at age 55 | NA | NA | - | Mitral valve endocarditis and prolapse; endocarditis | - | - | - | Valve abnormalities (aorta-valve and mitral-valve) | Mitral valve prolapse with mild regurgitation | - | - | - |
| **Published elsewhere** | doi: 10.1038/s41436-020-0850-1 | doi: 10.1038/s41436-020-0850-1 | doi: 10.1038/s41436-020-0850-1 | doi: 10.1038/s41436-020-0850-1 | No | No | doi: 10.1097/MCD.0b013e32834c4bb7 | doi: 10.1111/cge.12853. | doi: 10.1016/j.nmd.2020.09.002 | doi: 10.1111/1346-8138.12829 | doi: 10.1111/cge.12853. | doi: 10.1111/cge.12853. | doi: 10.1002/ajmg.a.30671 | doi: 10.3390/genes10110843 | No |
| **Patientnumber in article** | p6 | p7 | p8 | p17 | - | - | p3 | p3 | Patient from case report | Patient from case report | p6 | p2 | p2 | p1 | - |
| **Consent for publication** | Yes | Yes | Yes | Yes | Yes | Yes | Yes | Yes | Yes | Yes | Yes | Yes | Yes | Yes | Yes |
